# Preclinical development and evaluation of nanobody-based CD70-specific CAR T cells for the treatment of acute myeloid leukemia

**DOI:** 10.1007/s00262-023-03422-6

**Published:** 2023-03-17

**Authors:** Jiali Cheng, Tong Ge, Xiaojian Zhu, Jue Wang, Yuhao Zeng, Wei Mu, Haodong Cai, Zhenyu Dai, Jin Jin, Yongkun Yang, Guang Hu, Xia Mao, Jianfeng Zhou, Li Zhu, Liang Huang

**Affiliations:** 1 Department of Hematology, Tongji Hospital of Tongji Medical College, Huazhong University of Science and Technology, Qiaokou District, 1095 Jiefang Avenue, Wuhan, China; 2 Department of Internal Medicine, Cleveland Clinic, Akron General, Akron, OH USA; 3 IASO Biotherapeutics, Nanjing, China

**Keywords:** CD70, AML, Nanobody-CAR T-cells, Antigen density, Chidamide, Decitabine

## Abstract

**Background:**

Acute myeloid leukemia (AML) treatment remains challenging. CD70 was reported as a promising AML-specific antigen. Preclinically, CAR T-cell with single-chain-variable fragment (scFv) or truncated CD27 targeting CD70 has been reported to treat AML. However, various disadvantages including spontaneous exhaustion, proteinase-mediated loss of functional receptors, and high immunogenicity, limited its further application to clinical settings. Alternatively, the single-variable domain on heavy chain (VHH), also known as nanobodies, with comparable binding ability and specificity, provides an optional solution.

**Method:**

We generated CD70 knocked-out novel nanobody-based anti-CD70-CAR T-cells (nb70CAR-T) with two different VHHs for antigen detection. Next, we detected the CD70 expression on primary AML blasts by flow cytometry and associated the efficacy of nb70CAR-T with the target antigen density. Finally, epigenetic modulators were investigated to regulate the CD70 expression on AML cells to promote the functionality of nb70CAR-T.

**Results:**

Our nb70CAR-T exhibited expected tumoricidal functionality against CD70-expressed cell lines and primary AML blasts. However, CD70 expression in primary AML blasts was not consistently high and nb70CAR-T potently respond to an estimated 40.4% of AML patients when the CD70 expression level was over a threshold of 1.6 (MFI ratio). Epigenetic modulators, Decitabine and Chidamide can up-regulate CD70 expression on AML cells, enhancing the treatment efficacy of nb70CAR-T.

**Conclusion:**

CD70 expression in AML blasts was not fully supportive of its role in AML targeted therapy as reported. The combinational use of Chidamide and Decitabine with nb70CAR-T could provide a new potential for the treatment of AML.

**Supplementary Information:**

The online version contains supplementary material available at 10.1007/s00262-023-03422-6.

## Introduction

Acute myeloid leukemia (AML) is a group of heterogeneous hematological malignancies generated from clonal expansion of myeloid blasts without a tendency for mature differentiation [1, 2]. Allogeneic hematopoietic stem cell transplantation (HSCT) following induction chemotherapy is recommended as standard treatment for AML patients. In the past decades, with the advancement of therapy[3–5], the overall five-year survival of AML increased to 42.0 ~ 51.9% [6]. Nearly 50% of patients relapsed after HSCT from minimal residual disease and were eventually resistant to rescue chemotherapy [7, 8]. Therefore, more effective therapy for AML is urgently needed.

Adaptive transfer of engineered chimeric antigen receptor (CAR)-modified T-cells, has revolutionized the treatment of multiple types of cancers [9, 10]. Regarding AML treatment, CAR T-cell therapies were also in the spotlight, while the progression was slow [11–14]. One obstacle was the identification of suitable targets with the high-level expression on the AML blasts but expressed at absent or low-level on hematopoietic stem cells (HSC) [15]. Up to now, targets studied in AML treatment included CD33 [13], CD123 [11], CD44v6 [16], Lewis Y [17], CLL-1 [12], FLT3 [14], FR beta [18], and CD70 [19, 20]. The expression level of Lewis Y, CD44v6, CLL-1, and FLT3 were not as high as CD33 and CD123 on AML cells, but CD33 and CD123 were also expressed on normal HSC that could generate hematopoietic toxicity. CD70, a member of the TNF family, was recently reported to be an ideal target for AML given its up-regulation on AML blasts but absence on HSC [19, 20].

Another barrier was the design of the extracellular-antigen-binding domain of CAR. The most popular structure was the single-chain variable-fragment (scFv) which accommodates both heavy and light chains of antibody [20, 21]. Despite excellent binding affinity and specificity, various disadvantages have been recognized: (1) Some scFvs are prone to self-aggregation, and thus induce CAR clustering, generating tonic activation signaling, and eventually leading to T-cell exhaustion [22–24]. (2) Immunogenicity of murine-derived scFv [25, 26]. For CD70-specific CAR, truncated CD27 (tCD27) was also investigated and suggested that it had superior functionality than traditional scFv-based CAR [20]. However, matrix metalloproteinase-mediated cleavage of CD27 after engagement with CD70 results in reduced expression of the functional CAR on the T-cells, thus severely compromising the in vivo efficiency of tCD27-based CAR-T [21]. These drawbacks may limit the in vivo activities of 70CAR T-cells, and their further application to clinical settings. Alternatively, a fully-human single-variable domain on heavy chain (VHH) nanobodies with comparable binding ability and specificity is possibly a favorable choice.

In this study, we generated novel nanobody-CAR T-cells that contain two different extracellular-antigen-binding VHHs for CD70 detection (nb70CAR-T). Meanwhile, the *CD70* gene in T-cells was disrupted by the CRISPR-Cas9 system to avoid fratricide effects. We tested the tumoricidal activity of our nb70CAR-T against AML cells lines and primary blasts and their associations with the CD70 expression level, and verified the synergy effects of epigenetic modulators in the up-regulation of CD70 expression. Altogether, our data provided an in-depth evaluation of CD70 expression and CD70-targeting therapy for AML based on novel nb70CAR-T, which contributed to the initiation of related clinical trials.

## Materials and methods

### Cell lines and culture

THP-1 (Acute Monoblastic/Monocytic Leukemia), OCI-AML3 (Acute Myeloid Leukemia), Karpas299 (ALK-Positive Anaplastic Large Cell Lymphoma), MV4-11 (Acute Monoblastic/Monocytic Leukemia), K562 (Chronic Myelogenous Leukemia) and Lenti-X™293 T (human embryonic kidney cell) cell lines used in this study were purchased from American Type Culture Collection bank and ensured for mycoplasma-free before experiments. THP-1, OCI-AML3, Karpas299, MV4-11 and K562 were cultured in Roswell Park Memorial Institute (RPMI)-1640 medium (Gibco, USA) supplemented with 10% fetal bovine serum (FBS) (Gibco, USA). Lenti-X™293 T cells were cultured in Dulbecco’s Modified Eagle Medium (DMEM) (Gibco, USA) supplemented with 10% FBS. Cells lines were maintained in the incubator with 37 ℃ and 5% CO_2._ MV4-11 and THP-1 cells stably expressing firefly luciferase (ffLuc) were established by transduction with the lentivirus simultaneously encoding firefly luciferase and puromycin resistance gene, and cultured in a complete medium added with 1ug/mL puromycin (Gibco, USA).

### CD70 KO 70nbCAR T-cells production

T-cells were isolated from commercial peripheral blood mononuclear cell (PBMC) (Milestone® Biotechnologies, China) using anti-CD3 microbeads (Miltenyi, Germany), and were activated by microbeads (Dynabeads™ Human T-Activator CD3/CD28) (Gibco, USA). The CD70 gene was knocked-out by electroporation with Cas9 protein and small guide RNA (Genscript Biotech Corporation, China) using the Celetrix system. Eight hours post electroporation, CD70 knocked-out T-cells (KO T) were transduced with lentivirus encoding nb70CAR. After 24 h, nb70CAR-T and KO T-cells were centrifuged, resuspended in fresh culture medium, and sustained in a concentration of 1 ~ 2 × 10^6^ cells/mL. The complete culture medium was CTS™ OpTmizer™ medium (Gibco, USA) supplemented with 10% FBS and 200 IU/mL rhIL-2 (PeproTech, USA) and 100ug/mL L-glutamine (Gibco, USA).

Lentivirus encoding nbCD70CAR was generated by Lenti-X™293 T cells using psPAX2, p.MD2.G packaging plasmids and transfer-plasmid with Lipo3000 (Invitrogen, USA). Supernatants containing the released virus were collected 48 h post-transfection and concentrated by ultracentrifugation. Lentivirus was stored at – 80 ℃.

### Evaluation of CD70 KO efficacy

On day 7 of culture, the genomic DNA of KO-T was extracted by using a DNA blood mini-kit (Qiagen, Valencia, CA), and sent for Sanger sequencing of the CD70 gene. The data were analyzed using an online analyzing tool, ICE Analysis-Synthego (https://ice.synthego.com). The KO efficacy was calculated as the sum proportion of gene insertion/deletion.

### Binding affinity

VHH1 and VHH2 antibodies with rabbit IgG (rIgG) were manufactured by CHO-S cells (Chinese hamster ovary cells) and purified with protein A column. Bio-layer Interferometry (BLI) was employed to measure the binding affinity of VHH1-rIgG and VHH2-rIgG to CD70 antigen using an Octet96e system (ForteBio, Menlo Park, CA, USA) as previously described [27].

### Flow cytometry

The information on antibodies used in the flow cytometric (FCM) assay is included in Supplementary Table S1. CAR-positive cells were detected by anti-EGFR antibodies. IgG was used as an isotype for CD70 detection. For peripheral blood (PB)/bone marrow (BM) samples from patients, fluorescence minus one (FMO, IgG isotype was also added) group was set as a control.

Cell lines samples were washed by phosphate buffer saline (PBS) (Solarbio, China) supplemented with 1% FBS (Every green, China), stained with corresponding antibodies in 100 μL PBS with 1% FBS at room temperature for 20 min, avoiding light, and then were analyzed using Novocyte flow cytometry (Agilent, USA). In the detection of CD70 expression, Fc receptor blocker was used before antibody staining.

For PB /BM samples from patients, antibodies were incubated with whole blood samples containing approximately 2 × 10^6^ nucleated cells. After 20-min staining, red blood cells (RBC) were lysed using BD Pharm Lyse™ (BD, USA) and washed by PBS with 1% FBS. The prepared samples were processed by Canto™ flow cytometry (BD, USA) or Novocyte flow cytometry without delay.

For PB samples from mice, RBC was lysed, and mouse Fc receptor was blocked using 1:500 diluted Rat anti-Mouse CD16/CD32 (BD, USA). The samples were then ready for antibody staining, washing, and analyzing with MACSQuant Analyzer 10. The data were analyzed with FlowJo software version 10.

### Antigen density measurement

The CD70 expression density was measured using anti-CD70-PE (P-phycoerythrin) antibodies and PE fluorescent quantitation kit (BD, USA) per manufacture's instructions.

### Cytotoxicity assay

FMC assay or luciferase assay was exploited to determine the lysis of targets after incubation with nb70CAR-T at different effector-to-target (E: T) ratios. In the FMC-based cytotoxicity assay, 1 × 10^5^ target cells were labeled with 1 µM CFSE-FITC (Invitrogen, USA) at 37 ℃ for 20 min, and then washed with PBS to remove the residual CFSE. The labeled targets were then co-cultured with nb70CAR-T, or KO-T, or buffer (10% RPMI-1640) in 96-well U-bottom plates (Corning, USA) for 4 h or 24 h. Apoptosis analysis was conducted through PI and Annexin V staining. The proportion of necrotic (PI + /Annexin V-) and early apoptotic (PI-/Annexin V +) cells in CFSE-positive targets represented the cytotoxicity of nb70CAR-T or KO-T. In the luciferase assay, 2 × 10^4^ MV4-11-ffLuc cells were co-cultured with nb70CAR-T or KO-T in a 96-well flat bottom clear plate (Corning, CLS3610, USA) for 24 h. Luminescence representative for the remained live targets was quantified through a Synergy H1 microplate reader (BioTek, USA) and Steady-Glo® Luciferase assay system (Promega). The lytic percentage of targets was calculated by the following equation:

$${\text{Lysis}} \% = \frac{{{\text{luminescence }}\;({\text{buffer}}) - {\text{luminescence }}\;({\text{nb70CAR - T}})}}{{{\text{luminescence}} \;({\text{buffer}})}} \times 100$$

### Degranulation assay

Expression of CD107a (lysosome-associated membrane protein) on the membrane of 70nbCAR T-cells represents its degranulation process. 2 × 10^5^ target cells were co-cultured with nb70CAR-T (E: T ratio of 1) in a medium supplemented with 1:100 diluted anti-human CD107a antibodies and 1:500 diluted Monesin (BioLegend, USA) for 4 h. CD107a expression on CAR + T-cells was determined through FMC assay. The CD107a positivity on CD3 + CAR- populations represented the degranulation of KO-T. And only nb70CAR-T were set as a control to examine the auto-degranulation.

### Cytokine release assay

The level of IFN-γ, TNF-α and IL-2 were detected in the supernatants from 24-h co-cultures of nb70CAR-T or KO-T with targets at an E: T ratio of 1 in 10% RPMI-1640 medium using corresponding Enzyme Linked ImmunoSorbent Assay kits (NeoBioScience, China). IFN-γ in the serum of mice was detected by human IFN-γ kit (cisbio, USA) using HTRF® technology.

### Xenotransplantation assays

Animal study was performed under the approval of the ethical committee of Tongji Medical College. Six-week-old female NCG (NOD/prkdc-/- IL2Rg-/-) mice were obtained from GemPharmatech (China). THP-1-ffLuc (5 × 10^6^ cells per mouse) were injected into mice subcutaneously (subq.). Mice with successful tumor engraftment were randomized into three groups, and each group was infused with nbCAR70-T (2 × 10^6^ per mouse) or KO-T (2 × 10^6^ per mouse) in 200 µL PBS or just 200 µL PBS via tail vein. Tumor volume and body weight were measured almost every three days. Bioluminescence imaging was also conducted weekly. PB sample of mice was collected weekly through the submandibular vein for the detection of nb70CAR-T and the serum cytokine level. The protocol is summarized in Fig. 4A. The study was conducted with the consent of the Institutional Animal Care and Use Committee. All operations complied with the regulations of the Association for Assessment and Accreditation of Laboratory Animal Care.

### Primary AML sample processing and culture

The collection and use of clinical samples were approved by the ethical committee of Tongji Medical College, in accordance with the Declaration of Helsinki. The informed content of patients were obtained. PB or BM samples from newly diagnosed AML patients (excluding acute promyelocytic leukemia) were collected. PBMCs were isolated through density gradient centrifugation using Ficoll (STEMCELL, Canada) and cultured in 10% RPMI-1640 medium. Cytotoxicity assay and degranulation assay were conducted within 24 h after the isolation of PBMCs following the methods illustrated above.

### Decitabine and chidamide treatment

Decitabine (hypomethylation agent, HMA) and Chidamide (histone deacetylase inhibitor, HDI) were purchased from Selleck (USA) and Med Chem Express (USA), respectively, dissolved and stored according to the manufacturer’s protocol. For in vitro assay, the specified concentration of HMA and HDI alone or in together were added to PBS-washed cell lines or primary AML samples in 10% RPMI-1640 medium culturing for two days. A Dimethyl sulfoxide (DMSO) vehicle control was set. For *in mice* assay, female aged 6–8 weeks NCG mice were i.v with 1 × 10^6^ MV4-11-ffLuc cells. Five days later, mice with successful tumor establishment confirmed by bioluminescence imaging were randomized into three groups and intraperitoneally injected with 2 mg/kg/day HDI or 1.2 mg/kg/day HMA or DMSO for 4 consecutive days. At days 1, 3, and 5, 100 µL PB samples were collected and detected for the CD70 expression in MV4-11-ffLuc cells using FMC.

### Graphs and statistical analysis

GraphPad Prism 8.3.0(GraphPad Software, Inc., USA) was used for data analysis and graph plotting. Data were presented as mean values with standard deviation (standard error of the mean was used for data in animal study). For comparison between the two datasets, we used the Student *t* test or paired Student *t* test. Statistical significance was assumed when *P* < 0.05. All analyses were two-tailed.

## RESULTS

### 70nbCAR T-cells construction

**Figure1:**
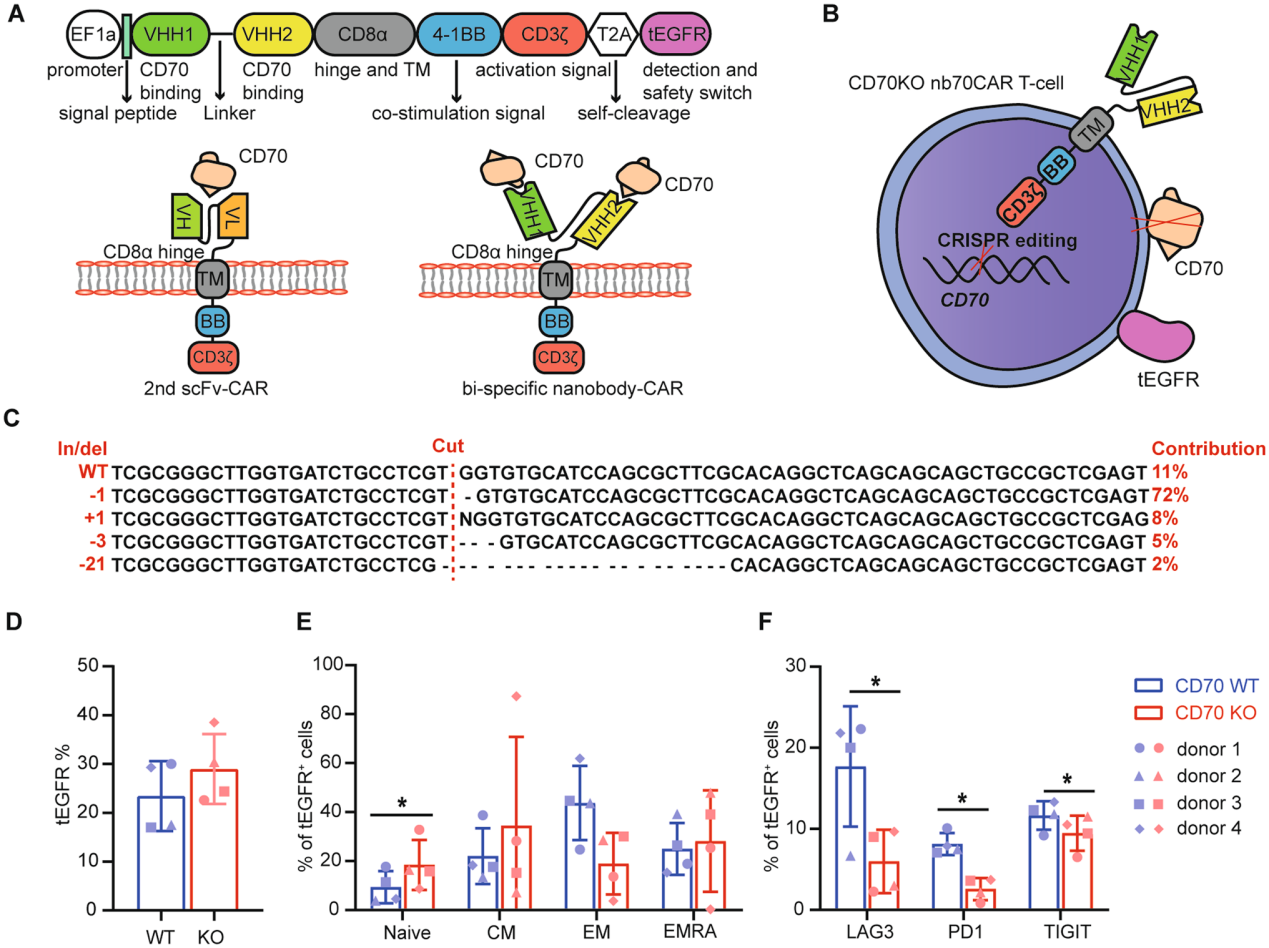
Construction and characteristics of CD70 KO nb70CAR-T. **A,** The novel nb70 CAR and traditional second generation (2nd) scFv-CAR construct. The function of each domain was also annotated. **B,** The schematic diagram of CD70 KO nb70CAR-T. **C,** Outcome of *CD70* gene Sanger sequencing of CD70 KO T-cells. + : insertion;  − : deletion; the number following “ ± ” refers to the numbers of bases. The gene editing efficacy is 87%. **D,** The transduction efficiency of nb70CAR in CD70 wild-type (WT) (blue column) and CD70 KO (red column) T-cells by flow cytometry using anti-EGFR antibody. No significance was identified between WT and KO groups using paired Student *t*-test (*n* = 4). **E,** T-cells memory phenotype of nb70CAR positive cells in CD70 WT (blue column) and CD70 KO (red column) groups by flow cytometry in four different donors, the biological source of PBMCs to produce nbCAR-T and KO-T. Naïve (CD45RA + CCR7 +), CM: central memory (CD45RA-CCR7 +), EM: effector memory (CD45RA-CCR7-), EMRA: effector memory cells re-expressing CD45RA (CD45RA + CCR7-). *P < .05, by paired Student *t*-test. **F,** Frequency of LAG3, PD1 and TIGIT in nb70CAR + T-cells in CD70 WT (blue column) and CD70 KO (red column) groups in four different donors. *P < .05, by paired Student *t*-test

CD70-specific VHH1 and VHH2, screened from an in-house fully human VH phage display library [27], were chosen as the antigen recognition domain. The binding affinity of VHH1 and VHH2 to CD70 antigen are 11.4 nM (K_on_ = 1.91 × 10^5^ M^−1^ s^−1^, K_off_ = 2.18 × 10^–3^ s^−1^) and 1.89 nM (K_on_ = 1.31 × 10^6^ M^−1^ s^−1^, K_off_ = 2.47 × 10^–3^ s^−1^). Our CAR is finally designed as *VHH1-linker-VHH2-CD8α-(4-1BB)-CD3ζ*, different from the common scFv-based CAR. Of note, tEGFR is contained in the CAR transfer plasmid and connected by T2A (2A self-cleaving peptides) as a safety switcher and detection marker (Fig. 1A, B). Importantly, CD70 is knocked out in our nb70CAR-T by implementing the CRISPR-Cas9 system before CAR lentiviral transduction (Fig. 1B). The gene editing efficacy determined by Sanger sequencing was around 87% (Fig. 1C). At 7 days of culture, the mean percentage of CAR + T-cells in the CD70KO group was slightly higher than the CD70 wild-type (WT) group (29.0 vs. 23.4%, *P* > 0.05) (Fig. 1D). Interestingly, at day 13 the CD45RA + CCR7 + naïve population in the CAR + pool in KO group was significantly higher than the WT group (9.4 vs. 18.4%, *P* = 0.048) (Fig. 1E). The KO group also expressed less PD1, TIGIT (markers of T-cell exhaustion), and LAG3 (markers of T-cell senescence) (Fig. 1F). In all, we successfully constructed a novel fully-human VHH_–_based tandem nb70CAR-T with the purpose of minimizing incidental immune escape mediated by some CD70 epitope loss. Knocking out CD70 in parental T-cells leads to higher-quality CAR-T product.

### nb70CAR-T tumoricidal activity in vitro

**Fig. 2:**
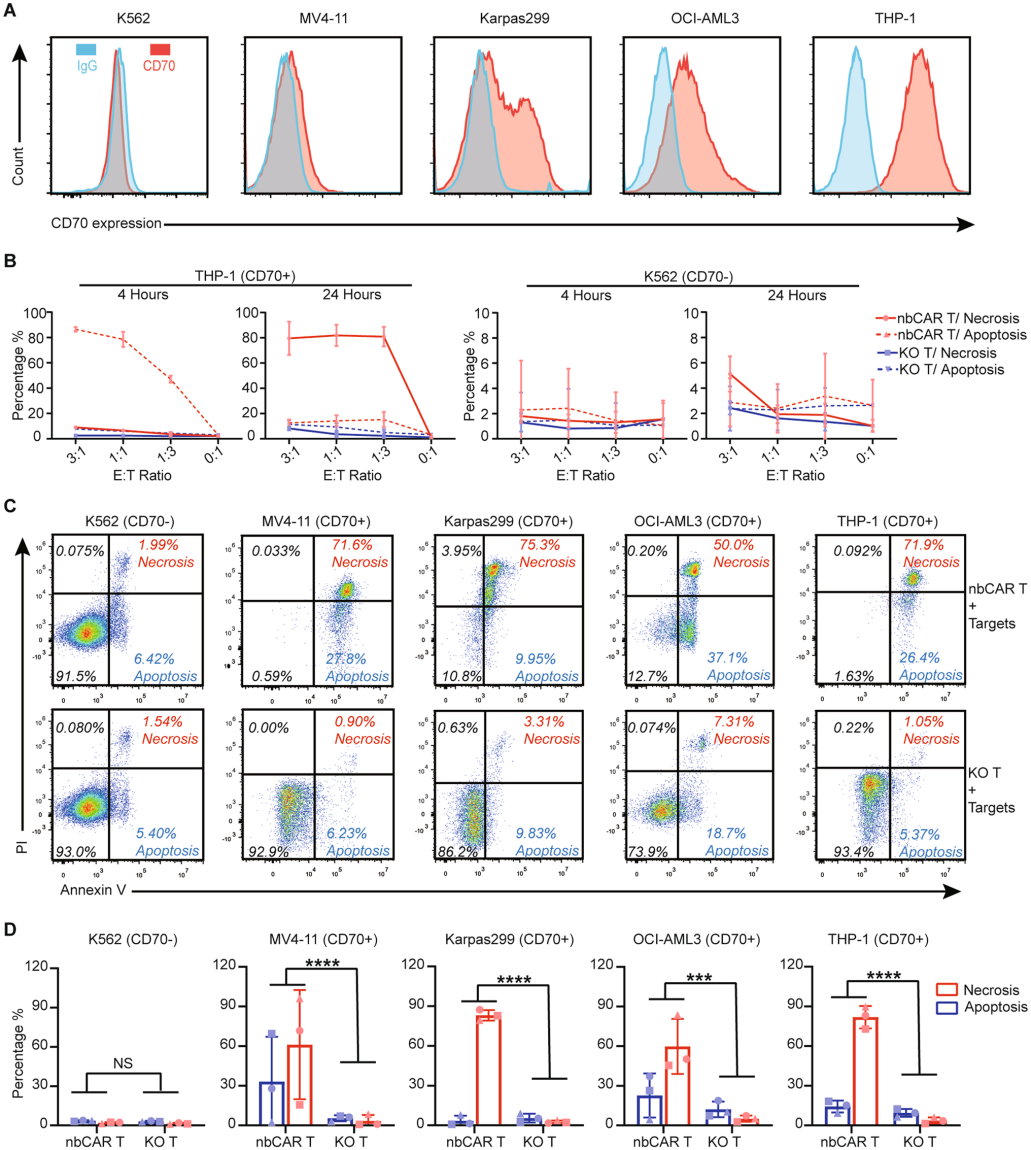
Cytolysis Efficacy of nb70CAR-T. **A,** Histogram of CD70 expression level/densities in the indicated tumor cell lines. **B,** The percentage of necrotic (PI + Annexin V +) and apoptotic (Annexin V + PI-) THP-1/K562 after incubation with nb70CAR-T or KO-T for 4 h or 24 h at different effector-to-targets (E: T) ratio as indicated in the *X*-axis. The red and blue lines were results from nb70CAR-T and KO-T, respectively. Solid lines and dotted lines represented necrosis and apoptosis, respectively. The mean value and SD from three biological replicates were shown. **C,** One representative dot plot of flow cytometry showing the population of necrotic and apoptotic K562, MV4-11, Karpas299, OCI-AML3, and THP-1 after 24-h co-culture with nb70CAR-T (the upper row) and KO-T (the lower row) at an E: T ratio of 1. **D,** Mean (SD) percentage of necrotic (red column) and apoptotic (blue column) K562, MV4-11, Karpas299, OCI-AML3 and THP-1 after 24-h incubation with nb70CAR-T or KO-T at an E: T ratio of 1, from three biological replicates. ***P < .001; ****P < .0001; NS, non-significance, by Student *t*-test

Cell-surface expression levels of CD70 in different human cell lines are quantified. Results demonstrated that CD70 expression was highest in the THP-1, followed by OCI-AML3, Karpas299, and MV4-11. CD70 was negative in K562 (Fig. 2A). The ΔMFI (difference to IgG)/positive rate of CD70 on THP-1, OCI-AML3, Karpas299, and MV4-11 cell lines were 392,413/98.4%, 68,845/63.7%, 32,085/33.7%, and 7,137/17.8%, respectively.

To investigate the functionality of our nb70CAR-T, THP-1 cell lines were co-intubated with nb70CAR-T and KO-T. In the nb70CAR-T group, after 4 h of co-incubation, 78.5% of THP-1 cells are in early apoptosis (PI-/AnnexinV +) and 6.6% are in necrosis (PI + /AnnexinV +) at an effector-to-targets (E: T) ratio of 1, and the cytotoxic capacity further strengthened after 24-h co-culture with 14.2%/81.8% of early apoptotic/necrotic targets (Fig. 2B). KO-T, quite the opposite, had little effect on targets in all conditions. Meanwhile, nb70CAR-T did not react with CD70-negative cell lines (K562), indicative of high selectivity. (Fig. 2B, C). We further evaluated the tumoricidal efficacy of nb70CAR-T in a wide range of human CD70-expressing cell lines. As shown in Fig. 2C (results from one representative experiment), after 24-h incubation with nb70CAR-T (E: T ratio = 1), 27.8%/71.6% of MV4-11, 10.0%/79.3% of Karpas299, 37.1%/50.2% of OCI-AML3 were in early apoptosis/necrosis. As controls, target cells still exhibited persistent viability after co-incubating with KO-T. We next validated the results in three different donors, the biological source of KO-T/nbCART, and identified similar results that nb70CAR-T had strong and specific cytotoxicity against CD70-expressing cell lines, with no effect on CD70-negative cell line (Fig. 2D). In addition, we compared the cytolytic capacity of our nb70CAR- T with the prior published studies (tCD27-derived CAR T [28], and scFv-based CAR T [19]). scFv-CAR T showed superior cytotoxicity against THP-1 in three biological replicates (Supplementary Fig. 1A). The nb70CAR-T demonstrated robust and comparable functionality with scFv-based CAR T-cells (Supplementary Fig. 1B).

**Fig. 3:**
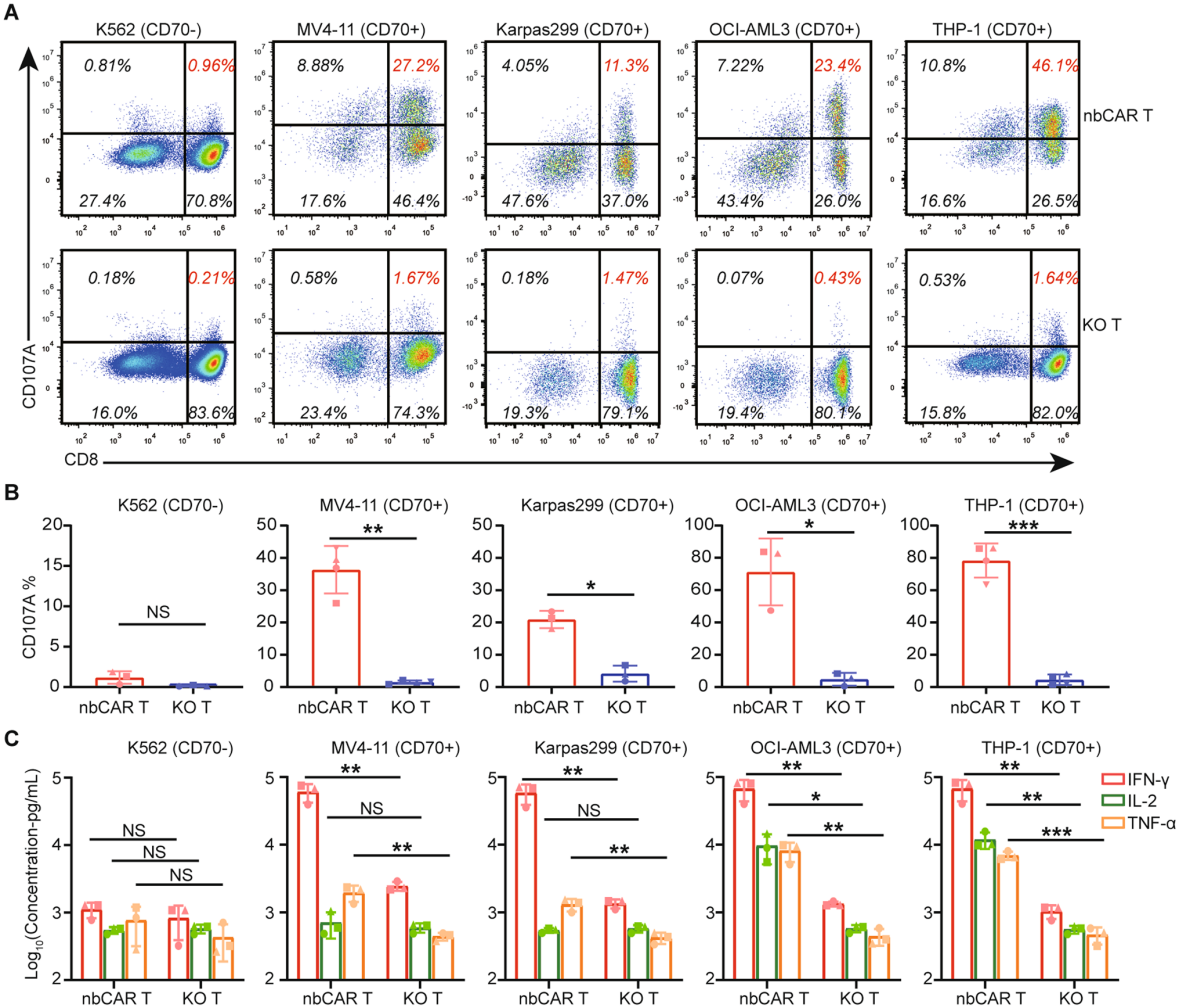
Activation of nbCAR T-cells under Stimulation of Tumor Cells. A and B, nb70CAR-T were incubated with K562, MV4-11, Karpas299, OCI-AML3, and THP-1 for 4 h, and CD107a expression in CAR + cells (nb70CAR-T) and CAR- cells (KO-T) were quantified. **A,** One representative dot plot of flow cytometry exhibiting CD107a expression in (CD8- and CD8 +) nb70CAR-T (upper row) and (CD8- and CD8 +) KO-T (lower row). **B,** Percentage of CD107a expression in CD8 + nb70CAR-T and CD8 + KO-T. Mean (SD) value from three or four different donors was shown, and *P < .05; **P < .01; ***P < .001; NS, non-significance, by paired Student *t*-test. **C,** IFN-γ (red column), TNF-α (yellow column) and IL-2 (green column) secretion by nb70CAR-T or KO-T after 24-h co-culture with the tumor cells. Results were from three different donors, *P < .05; **P < .01; ***P < .001; NS, non-significance, Student *t*-test

Activation of nb70CAR-T was simultaneously detected through CD107a and cytokine-release assay. The expression of CD107a on nb70CAR-T (CD8 +) was remarkably up-regulated after stimulations, with percentages of 27.2% (MV4-11 stimulation), 11.3% (Karpas299 stimulation), 23.4% (OCI-AML3 stimulation) and 46.1% (THP-1 stimulation). CD107a on CD8 + nb70CAR-T remained low upon K562 stimulation (1.0%). Plus, KO-T were barely stimulated by all cell lines (Fig. 3A). Consistent results were found using nb70CAR-T from three different donors (Fig. 3B). These indicated that nb70CAR-T were specifically activated by CD70-positive cell lines and underwent degranulation. In the cytokine release assay, the concentration of IFN-γ, IL-2, and TNF-α dramatically increased after 24-h incubation of nb70CAR-T with CD70-positive cell lines (E: T ratio = 1). nb70CAR-T produced the highest level of cytokines when incubated with THP-1 (67,635.5 pg/mL of IFN-γ, 11,929.8 pg/mL of IL-2, and 6,941.3 pg/mL of TNF-α, on average). Comparably, cytokines stayed low in both nb70CAR-T with CD70-negative cells (K562) and KO-T with all cell lines (Fig. 3C). The evidence of degranulation and secretion of immune-effective cytokines collectively proved that nb70CAR-T cells can be specifically activated by CD70-positive tumor cells.

### nb70CAR-T have potent tumoricidal activity in vivo

**Fig. 4:**
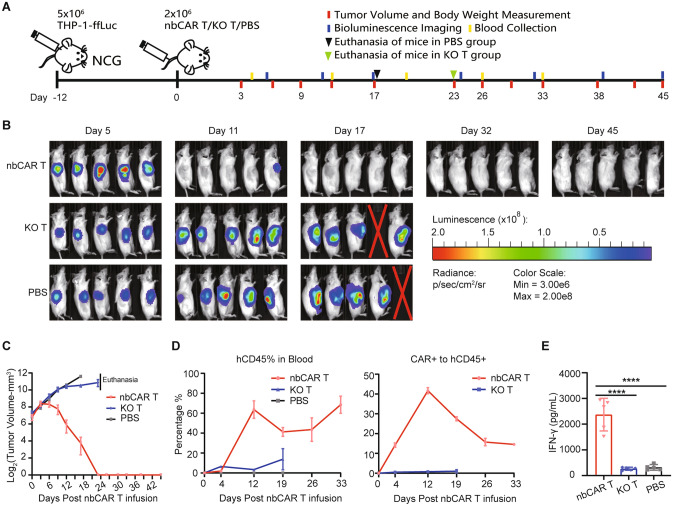
The anti-tumor efficacy of nb70CAR-T in mice xenografted with THP-1 cells. **A,** Experimental design: NCG mice were subcutaneously injected with 5 × 10^6^ THP-1-ffLuc cells on day -12. After successful engraftments of tumors, mice were randomized into three groups, receiving either: 2 × 10^6^ nb70CAR-T, 2 × 10^6^ KO-T or PBS on day 0. The tumor volume, body weight, bioluminescence imaging and blood collection were performed according to the schedule. Mice were euthanized when tumor volume was greater than 2000 mm^3^. **B,** Bioluminescent image of mice over time. **C,** Statistic of tumor volume in nb70CAR-T-treated, KO-T-treated, and PBS-treated groups over time. **D,** The mean (standard error) percentage of human CD45 + cells in the peripheral blood of mice, and the percentage of nb70CAR + T-cells to human CD45 + cells over time. **E,** Serum concentration of IFN-γ in nb70CAR-T-treated, KO-T-treated and PBS-treated mice at day 4. ****P < .0001, by Student *t*-test

We next examined the anti-tumor efficacy of our nb70CAR-T in mice model. The course is depicted in Fig. 4A. NCG mice were inoculated with THP-1-ffLuc subq., and treated with nb70CAR-T or KO-T delivered through the tail vein 12 days later (denoted as day 0) when tumor volume reached to approximately 130 mm^3^. nb70CAR-T exhibited robust anti-tumor effects *in mice* that the tumor began to shrink at day 3 and nearly disappeared within 2 weeks (Fig. 4B, C). While in the KO group and PBS group, tumor volume increased rapidly and exceeded 2000 mm^3^ at day 17 and day 21, respectively (Fig. 4B, C). All mice in the nb70CAR-T group survived at the end of observation (day 45), without tumor relapses, indicative of the potential long-term efficacy of nb70CAR-T. We also monitored the pharmacokinetics of nb70CAR-T in vivo. The percentage of hCD45 + cells (consisting of CD3 + tEGFR- T-cells and CD3 + tEGFR + CAR T-cells) in mice blood increased robustly and reached to 63.7% at day 12 in the nbCAR T group. While hCD45% remained low in the KO-T group (Fig. 4D). Moreover, nb70CAR + % in hCD45 + populations grew rapidly and reached an average peak value of 41.7% (SD: 3.1%) at day 12, then contracted and persisted at a mean level of 14.6% (SD: 1.2%) at day 33 (Fig. 4D). The level of immune effective cytokine, IFN-γ, in the serum of mice PB samples, collected ay day 4 in the nb70CAR-T group was significantly higher than that of the KO-T group (2,371.8 vs. 263.8 pg/mL, *P* < 0.0001) and PBS group (2,371.8 vs. 317.7 pg/mL, *P* < 0.0001) (Fig. 4E). These data indicated that nb70CAR-T can effectively eliminate CD70-expressing AML tumor cells *in mice* and possessed a favorable in vivo kinetic.

### nb70CAR-T tumoricidal effect toward primary AML blasts depends on CD70 expression

**Fig. 5:**
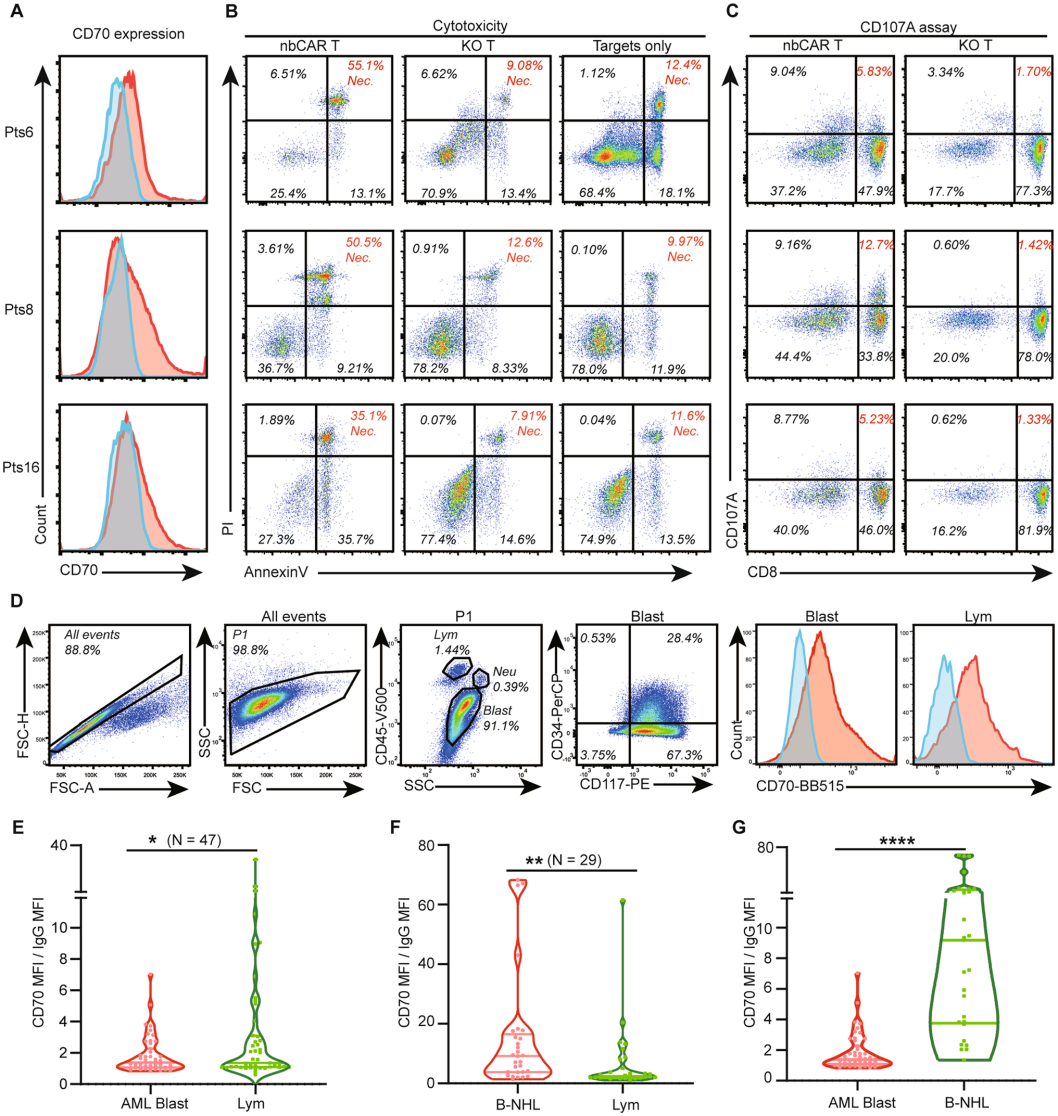
Response of nb70CAR-T to primary AML blasts and the CD70 expression. **A**, CD70 expression on AML blasts of the three patients (#6, #8, #16). **B,** Cytotoxicity of nb70CAR-T or KO-T against the three samples (#6, #8, #16). Dot plot of flow cytometry showing the population of necrotic (Annexin V + PI +) and apoptotic (Annexin V + PI-) targets after 24-h co-culture with nb70CAR-T or KO-T or buffer at an E: T ratio of 1. **C,** CD107a expression on nb70CAR + T-cells (CD8 + and CD8-) and KO-T (CD8 + and CD8-) after 4-h incubation with sample #6, #8 and #16. **D,** One representative sample showing the gating strategy of flow cytometry and the results of CD70 expression in AML blasts and lymphocytes. Blasts in the AML sample were identified by CD45 and SSC, and further checked for their CD117 and CD34 expression. **E,** Statistics of CD70 expression intensity, indicated by MFI of CD70 divided by MFI of corresponding IgG in AML blasts and the paired lymphocytes. *P < .05, *N* = 47, by paired Student *t* test. **F,** CD70 expression intensity in primary B-NHL tumor cells and the paired normal lymphocytes. **P < .01, N = 29, by paired Student *t*-test **G,** Comparison of CD70 expression in B-NHL tumor cells and AML blasts. ****P < .0001, by unpaired Student *t* -test

After the functional verification in vitro and in vivo, we started a preclinical investigation of our novel nb70CAR-T in the treatment of AML. We recruited 18 patients to examine the tumoricidal effect of nb70CAR-T on primary AML cells. Details regarding subtypes of AML, tumor burden, and genetic abnormality in those AML samples are reported in Supplementary Table 2. By our definition, it is considered that nb70CAR-T has a significant killing effect on primary tumor cells when the proportion of necrotic targets (PI +) in the nb70CAR-T group is at least 2 times higher (also no less than 10% absolute value increments) than the control groups (KO-T group or buffers only). In conclusion, a significant cytotoxic effect was observed in three samples (#6, #8, and #16) after 48-h incubation with nb70CAR-T at an E: T ratio of 1, (nb70CAR-T vs KO-T: 55.1 vs. 9.1%, 50.5 vs 12.6%, and 35.1 vs. 7.9%) (Fig. 5A, B). In the meanwhile, the CD107a assay revealed the rates of CD107a positivity in the CD8 + nb70CAR-T were 24.3, 8.6, and 7.5%, and the corresponding positive rates on CD8 + KO-T were 1.8, 1.6, and 2.2%, respectively (Fig. 5C), indicating specific activation of the nb70CAR-T. However, in the rest of the 15 samples, no similar killing effect was seen, nor was specific activation of nb70CAR-T (Table1, at the end of the manuscript). We found that the 3 samples that could activate nb70CAR-T all had a relatively high level of CD70 expression (Fig. 5A, Table 1). The ratio of the Mean Fluorescent Intensity (MFI) of CD70-FITC to the MFI of IgG-FITC was calculated to represent the expression intensity of CD70. The MFI ratios were 2.1, 1.8, and 1.6 in the 3 samples, and were no more than 1.4 in the remaining 15 patients. Regardless, there was recognizable consistency in the positive outcomes that the killing effect of nb70CAR-T depended on the CD70 expression level of tumor cells.

**Table 1:** CD70 Expression, Cytotoxic Effects, and Degranulation

Patient ID	Sample Type*	CD70 expression level (ratio)	Tumor burden (%)	△%Necrosis (post-stimulation)	%Necrosis increase rate	△%CD107a (post-stimulation)	%CD107a increase rate
**01**	BM	0.8	87	− 5.9	-0.5	1.9	0.8
**02**	BM	1.1	90	22.3	0.9	− 0.3	− 0.2
**03**	PB	1.0	92	3.1	0.3	1.2	0.9
**04**	PB	1.3	65	2.9	0.1	0.8	0.9
**05**	PB	1.4	86	− 11.1	− 0.6	− 0.1	− 0.1
06	**BM**	**2.1**	**85**	**42.7**	**3.4**	**7.5**	**2.3**
**07**	BM	1.2	85	7.1	0.8	0.9	0.6
08	**BM**	**1.8**	**52**	**37.9**	**3.0**	**24.3**	**8.1**
**09**	BM	0.9	82	− 4.8	− 0.3	4.2	1.7
**10**	PB	1.1	48	0.0	0.0	3.3	2.0
**11**	PB	1.0	95	− 1.8	− 0.4	0.1	0.0
**12**	PB	1.1	97	− 0.2	− 0.2	0.4	0.2
**13**	PB	1.3	81	11.3	0.4	1.6	0.3
**14**	PB	0.9	61	4.3	0.2	1.6	1.7
**15**	BM	1.1	88	− 12.4	− 0.2	0.7	0.8
16	**BM**	**1.6**	**82**	**23.5**	**2.0**	**8.6**	**5.4**
**17**	PB	0.9	41	9.5	2.4	2.3	2.0
**18**	BM	1.2	88	− 1.1	− 0.1	0.1	0.0

^*^Bone Marrow(BM); Peripheral Blood (PB)

CD70 expression level (ratio): MFI on AML blasts (CD70) / MFI of IgG (control);

△%Necrosis: % necrosis targets in nbCAR T-cells groups—% necrosis targets in control (KO T-cells or targets only);

%Necrosis increase rate: △%Necrosis / %Necrosis targets in KO T-cells groups or control (KO T-cells or targets only);

△%CD107a (post-stimulation): %CD107a positive in CD8 + nbCAR T-cells—%max CD107a positive in control (KO T-cells w/targets stimulation or nbCAR T-cells w/o targets stimulation);

%CD107a increase rate (post-stimulation): △%CD107a / %max CD107a positive in control (KO T-cells w/targets stimulation or nbCAR T-cells w/o targets stimulation);

Bold front: the three cases with significant nb70CAR-T responses

Encouraged by the apparent potential that when CD70 expression intensity exceeds 1.6, patients may benefit from nb70CAR-T therapy, we detected the expression level of CD70 on AML blast in more clinical samples to evaluate the clinical prospect of nb70CAR-T in the treatment of AML. BM or PB samples from 47 newly diagnosed AML patients (including the previous 18 patients) were evaluated for the expression level of CD70 on primary AML cells by flow cytometry. The gating strategy is depicted in Fig. 5D, and the mean expression intensity (MFI ratio) of CD70 on AML was 1.8, significantly lower than that of normal lymphocytes (3.7) (Fig. 5E). We further investigated the expression of CD70 on normal lymphocytes and its subsets T/B/NK cells in PB samples from 6 healthy donors. As shown in Supplementary Fig. 2, the mean positive rate on lymphocyte and T/B/NK is 5.3% and 6.1%/5.9%/5.3%. In the CD8 + DR + and CD8 + CD69 + T-cells subgroups (CD69 and HLA-DR are markers for T-cells activation), the rate is 6.3%/12.4%. Among the 47 patients, 19 cases (40.4%) of CD70 expression levels on AML blast exceeded the response threshold of 1.6 and the remaining patients had low CD70 expression levels and did not support nb70CAR-T treatment. The clinical information of these 47 patients is included in Supplementary Table 2. We tried to figure out factors associated with the CD70 expression level on AML blasts, but unfortunately, no significant parameters were identified, including the FAB (French-American-British) classification of AML, chromosome karyotype abnormality [translocation (8; 21), inversion (16), translocation (9; 11)], and specific gene mutations (*FLT3-ITD* and *DNMT3A*) (Supplementary Table 3). The small sample size of this analysis may conceal the regulators of the CD70 expression.

The expression of level of CD70 on AML blasts was not as high as expected. To verify our measuring system, we detected the expression level of CD70 on primary non-Hodgkin B-cell lymphoma (B-NHL) cells (generally acknowledged to have a high CD70 expression [29]) under the same condition. The mean/median CD70 expression level on B-NHL cells from 29 samples was 9.2/15.4, significantly higher than that of normal lymphocytes (*P* = 0.003) (Fig. 5C). In addition, we analyzed the expression level of CD70 mRNA based on data from the Cancer Genome Atlas Program and the Genotype-Tissue Expression project using an online tool GEPIA [30], and found that the level of CD70 mRNA in AML blasts (*n* = 173) was comparable to corresponding normal tissues (*n* = 70), but CD70 mRNA expression level in diffused large B-cell lymphoma (*n* = 47) was significantly increased compared with normal controls (*n* = 337) (Supplementary Fig. 3), consistent with the membrane expression level of CD70 protein identified in this study.

Altogether, nb70CAR-T tumoricidal effects toward primary AML blasts depended on CD70 expression. While variabilities existed in the CD70 expression on AML blasts, only a part of patients (40.4%) with high expression level was suitable for nb70CAR-T therapy. However, in view of the small sample size and simple investigations, more high-quality studies are needed.

### Epigenetic modulators facilitated the efficacy of nb70CAR-T by up-regulation of CD70 expression in AML blasts

**Fig. 6:**
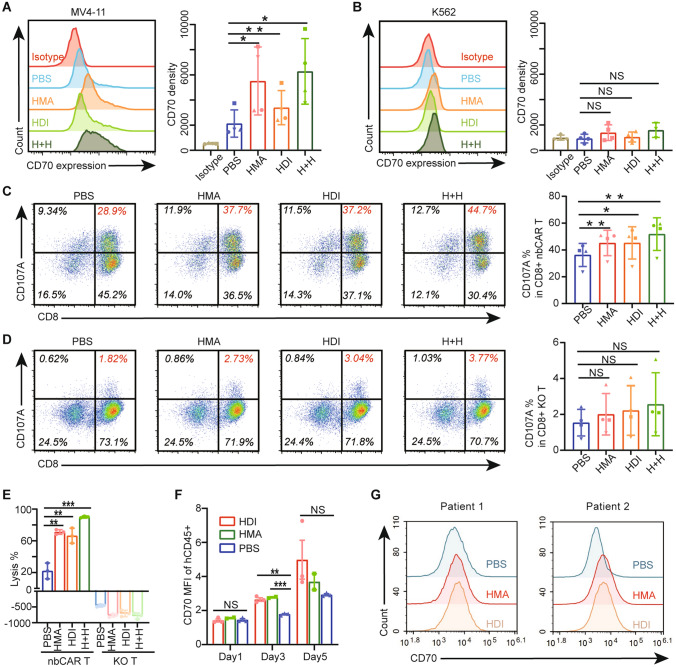
Enhancing CD70 expression facilitated the efficacy of nb70CAR-T. **A and B,** CD70 expression level on MV4-11 **(A)** and K562 **(B)** after 48-h co-culture with 0.3 µM Chidamide (HDI) or 0.3 µM Decitabine (HMA) or 0.3 µM Chidamide plus 0.3 µM Decitabine (H + H) or PBS. *P < .05, **P < .01, NS: non-significance, Student *t*-test, 3 ~ 4 independent experiments. **C and D,** Dot plot of flow cytometry displaying CD107a expression in nb70CAR-T (CD8 + and CD8-) (C) and KO-T (CD8 + and CD8-) (D) upon stimulation with MV4-11, HMA treated-MV4-11, HDI-treated MV4-11 and (H + H)-treated MV4-11. Comparison of CD107a expression in CD8 + nb70CAR-T (C) and CD8 + KO-T (D) between HMA and/or HDI treated groups and PBS treated groups, *P < .05, **P < .01, NS: non-significance, by paired Student *t*-test, using 4 data from different donors. **E,** Lytic percentage of MV4-11-ffLuc (PBS-treated, HMA and/or HDI-treated) after 24-h co-culture with nb70CAR-T or KO-T. Three technical replicates using one donor, **P < .01, ***P < .001, NS: non-significance, Student *t-* test. **F,** CD70 expression in MV4-11-ffLuc (human CD45 +) after 4-day intraperitoneal administration of 2 mg/kg/day HDI (*n* = 3) or 1.2 mg/kg/day HMA (*n* = 2) or PBS (n = 3) in NCG mice intravenously injected with MV4-11-ffLuc. **P < .01, ***P < .001, NS: non-significance, Student *t*-test. **G,** CD70 expression on AML blasts after 48-h co-incubation with 0.2 µM Decitabine (HMA), or 0.2 µM Chidamide (HDI) or PBS in two samples

Epigenetic dysregulation was a predominant character of AML, and hypomethylating agents (HMA) were reported to enhance the level of CD70 expression in the AML blasts [19]. We studied the impact of Decitabine (HMA) and Chidamide (HDI) alone or together on the CD70 expression in AML cell lines. In the MV4-11 (moderate CD70 expression) group, after 48-h treatment with 0.3 µM HMA, or with 0.3 µM HDI, or 0.3 µM HMA and 0.3 µM HDI, the expression of CD70 was 2 times, 2.75 times, and 4.7 times higher than PBS-treated groups, respectively (Fig. 6A). The up-regulation effect was also identified in the Molm13 cell lines (acute myeloid leukemia cell, moderate-high CD70 expression) (Supplementary Fig. 4). Data demonstrated that both HMA and HDI up-regulated the CD70 expression through promoting gene transcription, and the combination use of HMA and HDI can result in a stronger expression profile (Supplementary Fig. 5). However, neither Decitabine nor Chidamide influenced CD70 expression in K562 cells with negative CD70 expression (Fig. 6B), and THP-1 cells (Supplementary Fig. 4) with the baseline expression level being very high. The upregulation in turn facilitated the activation of nb70CAR-T. There was a significantly higher expression of CD107a in nb70CAR-T after co-incubated with HMA or HDI pre-treated MV4-11 cells (Fig. 6C). KO-T in all groups did not show any up-regulating expression of CD107a (Fig. 6D). The cytolysis efficacy of nb70CAR-T against MV4-11 was also  significantly improved after pre-treatment with Decitabine alone (× 3.2 times) or Chidamide alone (× 3.0 times) or combination (× 4.1 times) (Fig. 6E).

We further evaluated the effect of Decitabine and Chidamide on the CD70 expression of MV4-11 *in mice*. CD70 expression on MV4-11 cells remained unchanged 1-day post Deticabine or Chidamide treatment, but significantly increased to 1.56 times (Decitabine) and 1.49 times (Chidamide) on Day 3, and returned to baseline (the expression level of PBS-treated group) at day 5 (Fig. 6F). We also tested the induction of CD70 expression of Decitabine and Chidamide on primary AML samples. After 48-h co-incubation with 0.2 µM HMA or HDI, the CD70 expression on AML blasts in two samples was 1.2/3.5 times (HMA), and 1.3/3.6 times (HDI) higher than PBS-treated ones (Fig. 6G). These data collectively suggested that a low dose of Decitabine or Chidamide could promote CD70 expression on AML cells, and boost the therapeutic efficacy of anti-CD70 CAR T-cells. Combined use of Decitabine or Chidamide with nb70CAR-T in vivo was a promising strategy, worthy to be tested in AML patients.

## Discussion

In this study, we wish to highlight several aspects. First, novel CD70-specific CAR-T with two concatenated nanobodies (VHH1-VHH2) for antigen recognition was developed. The purpose of such a design is to minimize tumor escape mediated by some CD70 epitope loss. In anti-CD19 CAR-T therapy for acute B-cell leukemia, the frequency of relapse induced by epitope loss in CD19 was 10 ~ 20% [31]. However, further studies should be performed to identify the binding epitopes of the two VHHs, and to validate the functionality of VHH1-VHH2 CAR T-cells against tumor cells with some specific CD70 epitope loss that cannot be recognized by VHH1 CAR-T or VHH2 CAR-T or other published 70CAR- T. Besides, the *CD70* gene was knocked-out in our nb70CAR-T, which produced a final product with a higher proportion of naïve phenotype CAR T-cells and lower expression of PD-1 and LAG3. It was acknowledged that naïve CAR T-cells in the infusion product were correlated with improved long-term prognosis [32]. Higher expression of LAG3 and PD-1 in CAR T-cells products predicted impaired in vivo expansion, subsequently poor therapeutic responses in the chronic lymphocyte leukemia [33].

Second, CD70 seemed universally expressed in AML blasts, however, the level of expression varied exponentially. The expression intensity of CD70 on AML blasts in our dataset was an average of 1.8, with a SD of 1.2. In *Tim Sauer et. al.’*s research, CD70 expression in 136 BM samples of newly diagnosed AML patients was measured by immunohistochemistry. The distribution of H-scores representing the CD70 expression level was nearly in a symmetrical bell shape with a range of approximately 0 to 250, indicative of a large variation existing [20]. In *Meijia Yang et. al’s* study, based on the data in TCGA, the CD70 mRNA expression level was ranging from 2^–2^ to 2^8^ (calculated with RSEM, RNA-Seq by Expectation–Maximization), validating the fact that CD70 antigen expression level is characteristically heterogeneous [34]. Moreover, CD70 was also expressed on normal lymphocytes, imposing toxicity concerns in clinical settings. Although, no severe immunodeficiency was observed in the clinical trial of anti-CD70 therapy (NCT02830724, NCT03125577, NCT04662294), and CD70 is expressed only in parts of lymphocytes, especially activated T-cells (Supplementary Fig. 2), possible immunodeficiency induced by elimination of CD70 + lymphocytes cannot be totally excluded and long-term clinical observations are needed.

Multiple studies explicitly revealed that the efficacy of CAR-T critically depends on the target antigen expression density [35, 36]. Furthermore, the insufficient response against low-density antigen tumor cells leads to relapse or resistance to CAR-T therapy [37, 38]. Given the heterogeneous expression level of CD70 on AML blasts, it is important to identify a threshold density to reactivate anti-CD70 CAR-T. We evaluated the threshold based on in vitro cytotoxicity and degranulation assay using serials of primary samples. According to our cut-off value of 1.6 times MFI ratio (CD70-FITC/ IgG-FITC), only 40.4% of newly diagnosed AML patients fulfilled the standard and possibly benefited from nb70CAR-T therapy, much lower than expected. As CAR-T designs and experimental detections varied from study to study, it’s impossible to ascertain a definite and unifying expression threshold. Research on the development of novel CD70-specific CAR-T therapy for AML should characterize its own criteria of CD70 expression level to guide the inclusion of patients in future clinical trials. Besides, the functionality of CAR-T greatly depends on their structure. Optimal design can allow a more sensitive response threshold and therefore higher response rate among AML patients. nb70CAR-T had comparable functionality with the Cusatuzumab-derived CAR-T toward THP-1 cell line. Further comparison in primary AML samples will provide more valuable information on their therapeutic potency, worthy to be done in future.

Moreover, factors associated with CD70 expression need critical attention. Harper et al. [39] pointed out that the CD70 antigen was upregulated in various cell types possibly through activated T-cells after the administration of anti-CD70-CD3 bi-specific antibodies. It is in earnest to see whether the CD70 expression in AML cells will be up-regulated in the presence of 70CAR-T. Besides, CD70 was found to be upregulated in refractory or recurrent tumors, including glioblastoma [40] and mantle cell lymphoma [41], More in-depth researchers found that other conditions, including but not limited to tissue injury, autoimmune statuses (e.g., inflammatory bowel disease), hypoxia, proinflammatory cytokines (TNF-α and IFN-γ), and CD40L-CD40 signaling, can promote CD70 expression [42–44]. However, no related studies have been done in AML settings. We examined the FAB classification of AML, specific chromosomes, and genetic abnormalities, but no association with CD70 expression level were found. Such analysis based on large sample size and multicenter study was urgently required, which contributed to the understanding of the expression pattern of CD70, and then regulation of it.

Finally, to overcome the unresponsiveness imposed by low antigen density, we attempted to utilize epigenetic modulators to boost CD70 expression. Consistent with previous studies, CD70 expression was up-regulated when AML cell lines were exposed to decitabine, through a mechanism of reducing the methylation of CD70 promoter and enhancing the transcription of SP-1, the transcriptional factor of CD70 [19, 45]. Furthermore, we sought to determine if HDIs could enhance CAR-T function by increasing CD70 density in AML cells. Previous research on systemic lupus erythematosus contended that histone deacetylase was able to mute the expression of CD70 by deacetylating histone in the CD70 gene promoter region and enabling condensed chromatin structure [46]. Our data supported that Chidamide pretreatment of AML cells can significantly enhance the functionality of nb70CAR-T in an antigen-expression-dependent manner. Nowadays, cocktail therapies are the foundation management of hematologic malignancies. Different epigenetic abnormalities participated in the dysregulation of gene expression in AML, which supported the combined use of epigenetic modulators. The single-arm, phase 1/2 clinical trial showed the combination of Decitabine and Chidamide, together with chemotherapy, benefited the prognosis of adult patients with refractory/relapsed AML[47]. Also, our data is the first clear evidence that the combination of Decitabine and Chidamide generated a synergistic effect on inducing CD70 expression. However, the effects of HMA and HDI on the expression of CD70 in primary AML blasts need to be verified in a larger sample size. And the in vivo outcomes of HMA and HDI promoting nbCAR T-cells therapy remains to be validated. The detailed regulatory mechanisms of HDI and how it interacted with HMA, are warranted to be elucidated as well.

Altogether, our experiments contribute to a novel, high-functional, CD70-KO, nb70CAR-T. We characterized its required targeting density, which will guide the inclusion criteria for future clinical trials. The effectiveness of nb70CAR-T in AML patients refractory to standard therapy merits further investigation in clinical trials.

### Supplementary Information

Below is the link to the electronic supplementary material.

Supplementary file1 (DOCX 631 KB)

Supplementary file2 (DOCX 20 KB)

Supplementary file3 (XLSX 20 KB)

## Data Availability

The data supporting the findings of this study are available from the corresponding authors upon reasonable request.
