# Supplementary material for: Preclinical development and evaluation of nanobody-based CD70-specific CAR T cells for the treatment of acute myeloid leukemia
Source: Cancer Immunol Immunother. 2023 Mar 17;72(7):2331–46. doi: 10.1007/s00262-023-03422-6 (PMC10264288; doi:10.1007/s00262-023-03422-6)
Supplement: Supplementary file 1 — Supplementary file1 (DOCX 631 KB) [file 262_2023_3422_MOESM1_ESM.docx]

**Supplementary figure 1**


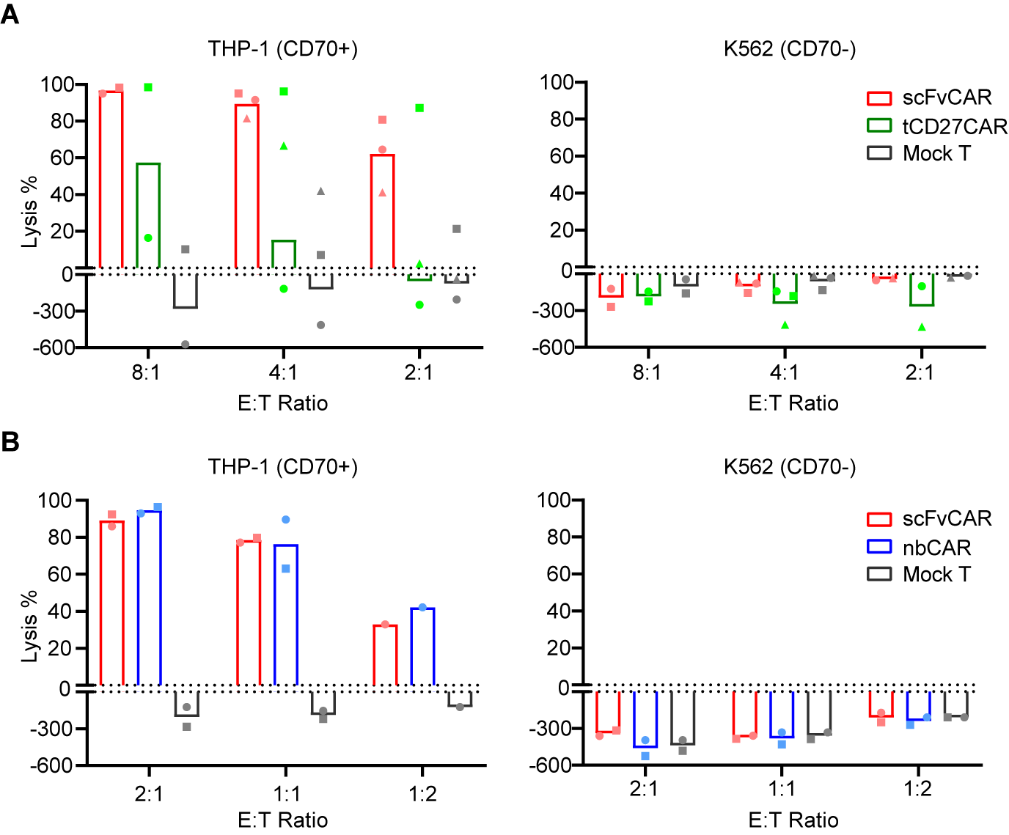


**Comparison of nb70CAR-T with prior published 70CAR-T.** **A,** scFvCAR was derived from Cusatuzumab (*Riether et al. 2020*), tCD27 CAR was from *Shaffer et al, 2011.* The cytolysis assay of the two CAR Ts against THP-1 and K562 were performed in three different biological repeats. **B,** The cytotoxicity of nbCAR-T and scFvCAR-T against THP-1 and K562. The data presented were the results of two technical replicates (performed in different days) using one biological source of T-cells.

**Supplementary figure 2**


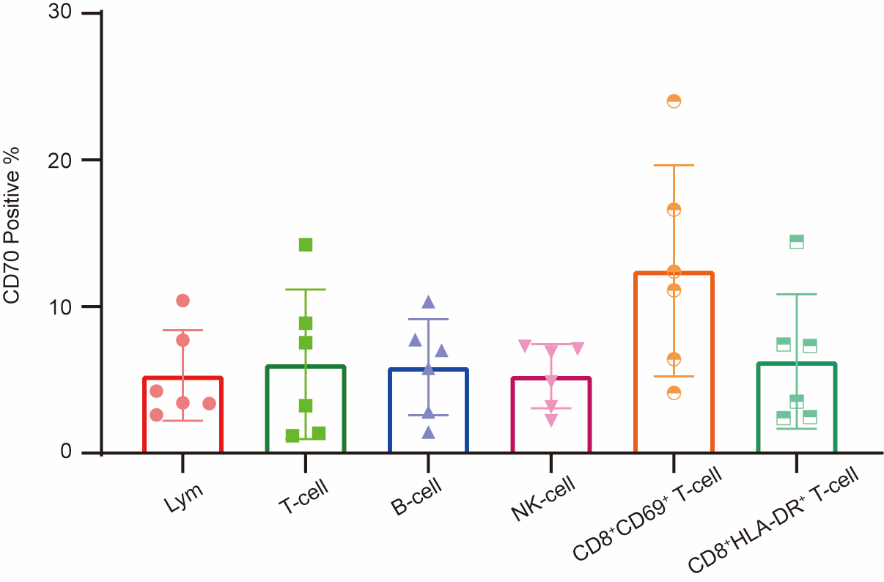


**Expression of CD70 on lymphocytes and some subsets.** The positive rate of CD70 on lymphocytes (Lym, gated by CD45 and SSC), T-cells (CD3+Lym), B-cells (CD19+Lym), NK-cells (CD3+CD56+ Lym), CD8+CD69+ T-cells, and CD8+HLA-DR+ T-cells in 6 healthy donors. The height of the column represents the mean value, and the error bar represent the standard deviation.

**Supplementary figure 3**


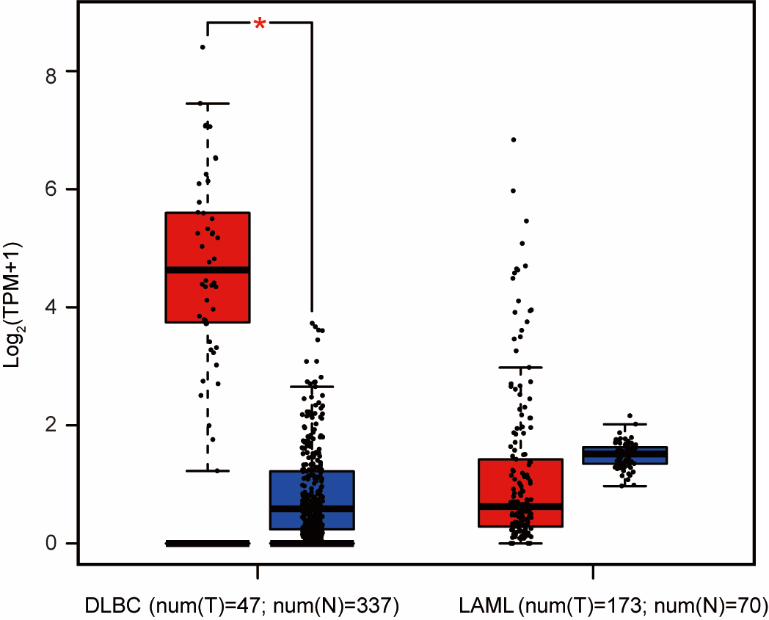


**CD70 mRNA level in the public database.** CD70 mRNA level in diffuse large B-cell lymphoma (DLBC) (N =337) and acute myeloid leukemia (LAML) (N = 173), and the comparison with the corresponding normal cells. Data were from TCGA and GTEx. analyzed by GEPIA, **P* < .01. TPM: Transcripts Per Million.

**Supplementary figure 4**


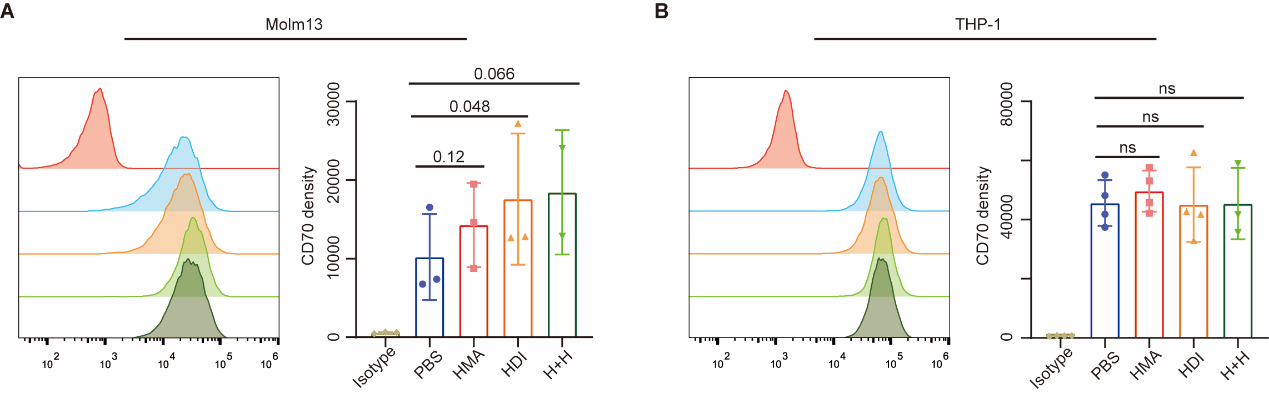


**Induction of Decitabine and Chidamide on the CD70 expression in Molm13 and THP-1. A and B,** CD70 expression level on Molm13 (**A**) and THP-1 (**B**) after 48-hour co-culture with 0.3µM Chidamide (HDI) or 0.3 µM Decitabine (HMA) or 0.3 µM Chidamide plus 0.3 µM Decitabine (H+H) or PBS. Isotype refers to IgG. NS: non-significance, Student *t*-test, 3 ~ 4 independent experiments.

**Supplementary figure 5**


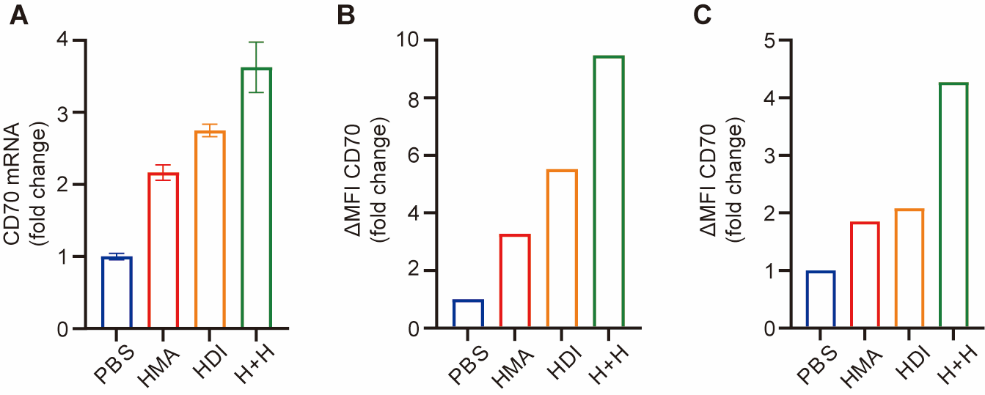


**Supplementary Figure 5.** **Preliminary study on the mechanism of epigenetic modulators regulation of CD70 expression.** The fold changes of CD70 mRNA expression level (**A**), total CD70 ΔMFI (CD70-BB515/IgG-BB515) (**B**) and cell membrane CD70 ΔMFI (**C**) in MV4-11 cells after 48-hour co-incubation with 0.3 µM HMA, or 0.3 µM HDI, or both 0.3 µM HMA + 0.3 µM HDI, relative to PBS-treated cells. The mRNA expression assay was conducted once with three parallel replicates. The CD70 ΔMFI assay was performed once. Total CD70 ΔMFI was determined by flow cytometry using cells fixed and permeabilized.
