# Supplementary material for: Preclinical development and evaluation of nanobody-based CD70-specific CAR T cells for the treatment of acute myeloid leukemia
Source: Cancer Immunol Immunother. 2023 Mar 17;72(7):2331–46. doi: 10.1007/s00262-023-03422-6 (PMC10264288; doi:10.1007/s00262-023-03422-6)
Supplement: Supplementary file 2 — Supplementary file2 (DOCX 20 KB) [file 262_2023_3422_MOESM2_ESM.docx]

| **Supplementary Table 1：Information of antibodies used in the study** | | |
| --- | --- | --- |
| Antibody | Clone | Company |
| anti-CD3 | OKT3 | BioLegend, USA |
| anti-CD70 | 113-16 | BioLegend, USA |
| anti-CD70 | Ki-24 | BD, USA |
| anti-CD8 | SK1 | BioLegend, USA |
| anti-CD107a | H4A3 | BioLegend, USA |
| anti-EGFR | AY13 | BioLegend, USA |
| anti-CD45 | HI30 | BioLegend, USA |
| anti-CD34 | 581 | BD, USA |
| anti-CD117 | 104D2 | BD, USA |
| anti-CD45RA | HI100 | BioLegend, USA |
| anti-CCR7 | 150503 | BioLegend, USA |
| anti-PD1 | EH12.1 | BioLegend, USA |
| anti-TIGIT | A15153G | BioLegend, USA |
| anti-LAG3 | T47-530 | BioLegend, USA |
| mouse-IgG1 isotype | MOPC-21 | BioLegend, USA |
| mouse-IgG3 isotype | J606 | BD, USA |
| Annexin V | / | BD, USA |
| PI | / | BD, USA |

| **Factors** |  | **CD70 low (<1.6)** | **CD70 high (≥ 1.6)** | ***P*-value** |
| --- | --- | --- | --- | --- |
| Age [median (range)] |  | 51 (25, 75) | 52 (0.9, 75) | 0.7507 |
| Gender [n, (%)] |  |  |  | 0.1367 |
|  | F | 13 (46.4) | 13 (68.4) |  |
|  | M | 15 (53.6) | 6 (31.6) |  |
| FAB [n, (%)] |  |  |  | 0.6132 |
|  | M0 | 1 (3.7) | 0 (0) |  |
|  | M1 | 7 (25.9) | 4 (23.5) |  |
|  | M2 | 4 (14.8) | 5 (29.4) |  |
|  | M4 | 3 (11.1) | 3 (17.6) |  |
|  | M5 | 12 (44.4) | 5 (29.4) |  |
| t (8;21) [n, (%)] |  |  |  | > 0.9999 |
|  | Neg. | 17 (94.4) | 16 (88.9) |  |
|  | Pos. | 1 (5.6) | 2 (11.1) |  |
| inv (16) [n, (%)] |  |  |  | 0.4857 |
|  | Neg. | 16 (88.9) | 18 (100) |  |
|  | Pos. | 2 (11.1) | 0 (0) |  |
| t (9;11) [n, (%)] |  |  |  | > 0.9999 |
|  | Neg. | 17 (94.4) | 17 (94.4) |  |
|  | Pos. | 1 (5.6) | 1 (5.6) |  |
| *FLT3-ITD* mutation [n, (%)] |  |  |  | > 0.9999 |
|  | Neg. | 18 (72.0) | 9 (75.0) |  |
|  | Pos. | 7 (28.0) | 3 (25.0) |  |
| *DNMT3A* mutation [n, (%)] |  |  |  | > 0.9999 |
|  | Neg. | 20 (80.0) | 9 (75.0) |  |
|  | Pos. | 5 (20.0) | 3 (25.0) |  |

**Supplementary Table 3: Association between CD70 expression level and clinical characteristics**

FAB, French-American-British classification of AML;

t, translocation; inv, inversion;

Neg.=Negative; Pos.=Positive;

Statistics: the Mann-Whitney test (for age), Chi-square or Fisher exact test for the others.
